# When is industry ‘sustainable’? The economics of institutional variety in a pandemic

**DOI:** 10.1007/s43253-023-00093-y

**Published:** 2023-04-24

**Authors:** Smita Srinivas

**Affiliations:** 1 Economics Department and Development Policy and Practice Group, Open University, Milton Keynes, UK; 2 Innogen Institute, Open University and University of Edinburgh, Milton Keynes, UK; 3 Department of Science, Technology, Engineering, and Public Policy (STEaPP), University College London (UCL), London, UK; 4 https://technologicalchangelab.org/

**Keywords:** Institutions, Institutional variety, Sustainable development, Health industry, Industrial policy, Technological change, B41, B52, O25, O33, L50, Q01

## Abstract

Industrialising economies today are characterised by a multi-level heterogeneity of customs, norms, guidelines, standards, regulations and other laws that provide the broad scaffolding and the technical context for industrial activity. This institutional variety (IV) leads to combinatorial challenges about which institutions are mixed and matched as technologies and sectors evolve. Gaps in evolutionary political economy and evolutionary institutional methods should explain when variety is ‘better’ for industrial development. Two health industry cases, oxygen production and Ayurveda, have come into the pandemic spotlight under high demand and high uncertainty, by patients, state, firms, experts and other stakeholders. Both cases reflect markedly different types of institutional variety with implications for manufacturing and services. A debate of sustainable industrial policies (SIPs) thus requires attention to institutional variety (IV) and a future agenda on healthcare.

## Institutional variety (IV) and industrialising economies

Under what conditions is industrial policy sustainable? Can the pandemic’s real-world industry response point to how sustainability might emerge in industries?

A wide economics and economic development scholarship now recognises industrial development as an institutional process of transformation driven by distinct technological capabilities. This acknowledgment that there may be various ways to achieving industrial development is partly recognised in policy parlance by the SDG 9 (Industry, Innovation and Infrastructure). UNIDO (2022) for example, defines industrial response during COVID-19 as central to the context of SDG 9. “Findings documented in the report strongly reaffirm the centrality of Sustainable Development Goal 9 (SDG 9) to the achievement of the 2030 Agenda for Sustainable Development” (UNIDO 2022, xi), and goes further to define SDG 9:

Sustainable Development Goal No. 9 (SDG 9):

Build resilient infrastructure, promote inclusive and sustainable industrialization and foster innovation. This goal promotes raising industry’s share of employment and GDP by 2030, integrating small-scale industrial and other enterprises into value chains and markets, upgrading infrastructure and industries with greater resource-use efficiency, using clean and environmentally sound technologies and industrial processes, boosting scientific research, upgrading technological capabilities and encouraging innovation (UN 2015) (UNIDO 2022, xviii).

This paper argues that a significant institutional variety (IV) of industrial customs, norms, guidelines, standards, regulations and other laws, exists in industrialising contexts. In principle, there are different ways of constituting, combining and carrying out such economic activity. Micro-level industrial and technology adjustment cannot always replicate a European or US template, and this is well recognised in economic development analysis. However, it is less well recognised that while different ex-ante institutional combinations are in principle possible, only some combinations of institutions are technologically possible and industrially sustainable in practice. This combinatorial open-endedness, yet specificity, requires more explicit attention (Srinivas 2018, 2020, 2021a, b).

The pandemic experience has underscored these gaps of understanding nations and industries’ responses. Wealthier industrialised countries and their health systems have experienced major economic and health hurdles. In the first full year of the pandemic, 2020, world gross domestic product (GDP) dropped by 3.3 percent, an estimated loss of 255 million full-time jobs, and an expected additional 97 million people into poverty (UNIDO 2022, 4). Industrial impact was widespread and adaptation mechanisms wide: Survey data during COVID-19 of a sample of firms in 26 Developing and Emerging Industrial Economies (DEIEs) small and medium enterprises (SMEs) show clear impact from the pandemic within every size category, and service sectors, firms or industries vulnerable to COVID-19. Furthermore, SMEs may experience an impact 10 times or more what larger firms experienced, and after the height of the pandemic, a drop in sales 14 times higher than larger firms in the ‘robust’ industry category (UNIDO 2022, 5).

For some industrialising nations in particular, pandemic and war geopolitics outside their controls have required new institutional combinations to maintain essential commodity and energy supply chains and urgent focus on indigenous technology development and domestic investment sites. Despite technology transfer barriers for the last 40 years in domains such as semiconductors, nuclear, aviation, aerospace (satellites and rocketry), food, chemicals and biotechnologies, investment has risen in Asian economies as investors (domestic, overseas migrants, institutional) all seek stability. India as the fastest growing economy today and the world’s fifth largest economy, with World Bank growth forecasts of 6.9%. It has seen a 13% rise in FDI with digital sector dominance across multiple sectors and a record inward remittance for a single country of $100 billion expected in 2022 (World Bank 2022). Given China’s 4% rise in 2020, Chinese political disruptions and pandemic shutdowns in 2021–2022 and the continuing Russia-Ukraine war, both China and India seek investment, growth and lend urgency to ‘sustainable’ industrial policy reforms. Furthermore, industrialised economies of Asia have also participated in global supply chains and acted as suppliers to other non-Asian industrialised economies during the pandemic (Falk et al. 2021).

Data also shows that during COVID-19, ‘sustainability’ of industries and their regional impact is correlated to the adaptation to severe drop in demand, access to resources (including labour-intensive goods and services since people were under lock-down or ill) and management of logistics bottlenecks (including national border closings). Table 1 indicates industrial response as “robust” or “vulnerable”.

**Table 1:** Vulnerability of industries to COVID-19

Industry type 1 “robust” Comparatively low impact, or strong negative impact but rapid recovery	Industry type 2 “vulnerable” Strong negative impact, unable to recover quickly
Essential goods (food and chemicals, but also paper) Industries that faced increased demand due to the health emergency (pharmaceuticals, computers and medical equipment) Capital-intensive high-tech industries that managed to bounce back swiftly from the initial impact (machinery and electrical equipment)	Labour-intensive industries (apparel, leather, textiles, furniture, other manufacturing) Capital-intensive industries affected by cross-border containment restrictions (motor vehicles, other transport equipment, petroleum)

Source: UNIDO (2022)

This industrial data does not however clearly resolve which combination of institutions (norms, customs, guidelines, standards, regulation, laws) emerges as resilient, but it can accept that societies may move toward different types of combinations that, at least for a while, serve them well in building technological capabilities during and despite the pandemic. Moreover, only some countries have had ‘sufficient’ technological capability and institutional adaptability to manage trade shutdowns and domestic response. Only some institutional combinations are thus ‘sustainable’, i.e. adaptable, resilient, or less costly in health, ecological, or financial terms across time. These institutional adjustments in some industrialising economies have also been possible under shorter time scales than might have been expected. Regions, industries, firms and workers are therefore axes that can throw some light (if not full conclusions) on the effects by the end of 2021 (see UNIDO 2022 Report summary).

Thus, while it appears that some ‘developing’ countries managed better ‘than expected’, these responses require more micro and meso-level context to study and evaluate. COVID-19 has also required substantial (and “robust”) health industry response itself, creating technological investments and generating new science and manufacturing pressures.

The structure of this paper is thus as follows.

Section 2 lays out the challenges of the combinatorial approach to institutional variety in evolutionary political economy and why it is essential to debates on sustainable industries. Section 3 addresses two co-existing health sector cases in India that saw sharp demand and uncertainty during the pandemic (1) Ayurveda and (2) oxygen production and delivery, to analyse the nature and degree of their institutional variety and ‘industrial’ facets. Section 4 presents preliminary observations of the two cases. Section 5 discusses ecological and economic facets of SIPs in health to connect institutional variety and industrial sustainability. Section 6 concludes with implications for evolutionary political economy and industrial policy.

## The combinatorial challenge

Evolutionary political economy (EPE) as a critical sub-field of economic theory has advanced both evolutionary and institutional (E-I) approaches. Many E-I methods have in common that they recognise variation in economic activity and use combinatorial lenses (Elsner 2012). However, the systemic basis for comparison between different theoretical and method frameworks within EPE remains unclear (Dolfsma and Leydesdorff 2009; Safarzynska and van den Bergh 2010; Elsner 2012). Srinivas (2020) argues that economics is a weak science in this sense, both the discipline and EPE lacking a systematic intra-paradigmatic approach to institutional variety (IV), gaps exacerbated by a narrow historical industrial focus. These gaps remain irrespective of separate ‘external’ problems of EPE with ‘mainstream’ economics. One explanation for the gap is a focus on selection mechanisms while lacking a consensus on how, methodologically, to address variety (Safarzynska and van den Bergh 2010, 363–364). Another, as this paper’s cases present, is the evolution of industrialising economies and their variety.

A theoretical focus on institutional variety (IV) has particular consequences for any study of ‘development’ especially the building of technological capabilities in industrial transformation (Srinivas 2020, 2021a). Within the Varieties of Capitalism (VoC) literature and the French Régulation approach, institutional variety and their combinations is referred to as “institutional complementarity” (see Hall and Soskice 2001; Amable 2000). However, the VoC scholarship leaves unexplained crucial development issues about inference and judgement on building technological capabilities or revealing the ‘system’s’ combinatorial basis for industrial development (Srinivas 2020, 2021b; Gerschenkron 1962; Amsden 2001). That variety matters is now recognised in EPE: “Evolutionary approaches have not been completely standardised yet as to their methods, giving rise to a profusion of answers to address the questions of dynamics, process, history, novelty, or disruption” (Safarzynska and van den Bergh 2010, 363–364). In this sense, institutional variety in *industrialised* economies reflects a narrower spectrum of a potential industrial ‘system’, an *ex-post* explanation for whom development paths and innovation strategies of those *industrialising* today are not reflected (see Arocena and Sutz 2000; Srinivas and Sutz 2008; Srinivas 2021a). Thus, countries will differ in their dominant institutions and organisations essential to building technological capabilities in agriculture, manufacture or services and also in the tensions of customs, norms and standards of authority by which practices, routines and system inclusion occurs. This conflict of sub-systems has been well recognised in older development political economy literature but their micro-level repercussions including positive adaptability are yet underdeveloped. Furthermore, digital transformation is accelerating the simultaneous automation and building of new cross-sectoral industry ties, making the elaboration of institutional variety across industries even more important. At its simplest, ‘sustainable industrial policies’ (SIPs) could serve as a selection device or even a residual variety of an industrial system’s evolution.

The pandemic’s effects and health responses have also to be situated amidst global trade stoppages and thus a reduced IV since global supply chains force-fit trade into harmonisation and homogenisation of firm-level strategies, requiring certification of specific technical standards and compliance with supplier business rules. Much smaller firms do experience diverse learning strategies and technology investments but, being at the margin, must weigh the benefits of different types of costly integration into these global supply chains. Labour cost advantages for these small firms will not last, and a combination of regional concentration trends and selective integration policies is needed within industrial policy design to assist them (see Kaplinsky, Wilkinson, Falk et al. 2021). Sustainability in well-studied ‘value chains’ may require more attention to how global firms train suppliers to meet global sustainability standards (Görg and Greenaway 2004; Schiller 2018) or transfer costs to those outside.

Mainstream economics suggests that industrial policy is superfluous or harmful based on market failure and government failure arguments. But partial or full state ownership of industrial assets may still be ambiguous on which regulation and incentives work best, even under well-accepted global institutional combinations such as patent law or common organisational forms such as cooperatives or R&D laboratories. Thus, unlike equilibrium trade approaches, evolutionary, institutional (E-I) concepts have greater appetite and conceptual tools for analysing institutions and the networks of stakeholders whose coordination is complex or organisational form is fluid (Srinivas 2012; Dolfsma 2019; Dolfsma and Mamica 2020).

Such variety has been visible in prior periods of today’s industrialised nations. For instance, institutions seen as sustainable or ‘alternative’ were visible in the norms and customs of workmanship and manufacturing in the original institutional economics or ‘American institutionalism’. In European industrial development, these include specific protections of knowledge, resulting in unique organisational and institutional forms: e.g. guilds and secrecy laws. Similarly, American institutionalism, Régulation and neo-Schumpeterian traditions have broadly recognised some types of variety and combinations (e.g. corporate R&D labs combined with intellectual property in particular ways). Thus, whether complementary institutions or hierarchy are useful explanations for variety requires much further study (see Magnin 2018, Loewen 2022 on industry, ‘national health systems’, e.g. Chataway et al. 2007). Specific methods can further reveal the tensions of argument (e.g. heuristics and taxonomy, Srinivas 2020, 2021a).

Similarly, crisis-induced institutional adjustments such as the post-1970s oil shocks or 'Washington Consensus' impact on structural adjustments generated concerns of too-rapid transfer of specific institutional bundles that were previously considered robust in the industrialised ‘North Atlantic’ economies. Furthermore, technological capabilities and industrial fortunes in Asia have not followed a single economic trajectory. This also helps to situate taken-for-granted ‘universal’ European or US models for Asia or other cultural and regional contexts when nation-state political exigencies influence what institutional combinations are possible in specific industries as viable ‘development policies’ (Andriesse and van Westen 2009; Srinivas 2018). Within EPE traditions also, there are also important reasons to argue for more attention to variety. For neo-Schumpeterians, IV is central to creativity (e.g. Saviotti 1996) but lacks a framework to explain when variety and its combinations result in ‘good’ development or industrial policy design less fragmented (Srinivas 2020; Papaioannou and Srinivas 2019). This gap needs attention since variety and fragmentation appear as essential features to the business cycle and innovation (Courvisanos 2009; Robert and Yoguel 2016).

Combinatorial approaches thus lend themselves to various scale of analysis: the products, technologies, techniques and instruments, and sub-sectors and industries, aiding ‘history-friendly’ studies and their policy context (e.g. Malerba et al. 2008), but also to customs, norms and standards of services, practices and products within organisations. In pharmaceutical history, for example, product and process patents dominate the choices firms make, but crucial are public procurement rules and the Current Good Manufacturing Practice (cGMP) technical standards in forcing firms to establish fixed organisation routines for process chemistry, scale-up and manufacturing, with clear quality and safety standards affecting diagnostic services. Specific industries may also have regional features (e.g. petrochemicals) and industrial processes (e.g. chemical engineering) which constrain the production possibilities of health industries and explain the national rise of synthetic pharmaceuticals and specific consumer goods based on chemical synthesis in Germany and the US. Therefore, institutional variety may shrink or strengthen in unique knowledge and proprietary trajectories (the Bayh-Dole Act, USA, see Hodgson 2007, Achilladelis and Antonakis 1992).

Therefore, a real-world problem better guides the understanding of models of selection and combination. Firms cannot immediately be conceived in a heterogeneous system without some type of heuristic through which we assess cohesion and convergence (Srinivas 2021a, b, c). Technologies can be developed and deployed in diverse ways because of the internal capabilities of firms but these are developed in a specific industry’s institutional context which evolves, sustainably or not (Dolfsma and Mamica 2020). The “Fit” of policy therefore matters to the institutional context:

An industrial policy that is not successful targets firms or firm activities that do not fit the circumstances suitable for that policy. A policy’s effects on the economy, to wit, depend on the circumstances under which it is implemented. In other words, fit between circumstances and dimensions of a policy is important to explain the outcomes of a policy (Ibid., 350).

Thus, institutional variety might be culled (selected for or against) toward innovation and competition between firms, by better honed (“sustainable”) industrial policy design of actual regulation, procurement, competition policies. Thus, the clarification of the inductive-deductive relationship through mixed methods is perhaps the most promising way to capture the extent of institutional variety, the nature of cohesion and customisation and the nature of convergence of the sub-sector or industry (Srinivas 2020, 2021a,c). Intermediate heuristics can thus develop taxonomies of country and industry histories, such as a co-evolving ‘institutional triad’ of production, demand and delivery through which more precise hypotheses can be generated (e.g. Srinivas 2012, see also Amable 2000 on 'social systems of production and innovation'). For industrialising democracies in particular, a much smaller sub-set of industrial history, technological capabilities are thus contingent paths and analytical cases in a wider political economy.

## An exploration of IV in two health industry cases

In engineering, “sustainable” manufacturing implies organisational or engineering practices best applied to routine and standardised manufacturing of commodity products, or longevity-focused resource-use of materials, niche areas of design and consumption, and where production processes and energy intensity and sources are newly scrutinised. But “sustainable” production systems in the wider ecological sense will have to source, use, reuse and recycle in minimally harmful ways and require financial systems that can respond and reward innovation that makes them even less harmful to natural resources. This ‘do no harm’ has to move beyond engineering and boost human health, not just efficient use of resources. ‘Sustainability’ concerns of manufacturing are thus more precisely concerns of adaptability to changing climactic conditions as well as variability of knowledge constraints within specific organisations and their standards (e.g. quality, resource-use, safety). Evaluation criteria may also create hurdles for technology integration (Mukherjee 2021). Thus, rather than universal propositions about industrial development, micro-cases may define which practices, routines or types of production (products, services) undergird its sustainable scale and scope.

This section contributes 2 Indian health industry cases. Three total cases (2 here) were presented and reviewed in two rounds of multilateral expert meetings and several prior consultative discussions. A separate ongoing 4-country cancer care project in which the author was involved during the pandemic evaluated similar concerns of Indian health industry adaptation and innovation. Using secondary data sources in a rapid timeline of pandemic change, the paper draws from scholarly articles, newspaper reports, interim published clinical cases, legal filings and daily published COVID-19 trackers.

The first case (Ayurveda) reflects a different scientific paradigm with contemporary clinical, manufacturing, and regulatory challenges. The second case (Oxygen) has both industrial scale production and medical grade delivery systems. Both involve different standardisation and certification systems and feed into multiple industries. A fuller research project is required after the pandemic.

### Case 1 Ayurveda: the industrial scaffolding of “traditional medicine”

Ayurveda, a “traditional medicine”, focuses on longevity and responsibility, through the lenses of quality, healthy living, often combined with yoga (the ‘Asthtanga’), a framework of ‘authenticity’ of ancient yogic practices and authority systems that are embedded in the wider norms and regulations of its society. Crucial complementary features on spirituality, right action, one’s nature, lifestyle emerge from the Vedic roots of Hinduism (‘Ayur’ life and ‘Veda’ Knowledge), and philosophical and logic systems, which are built into Hindu lifestyle and rituals and those of other *dharmic* philosophies such as Buddha Dharma and Jaina: e.g. *Dinacharya* (daily routines), Ritucharya (seasonal routines) including self-diagnostic and diagnostic *doshas* and corrective measures from appropriate work, time of day, harnessing one’s *gunas* and the place and measure of one’s freedom from one’s mind (see also Frawley and Lad 1994; Lad 2002). Ayurveda is thus as lifestyle, rooted in traditional Indian foods, seasonal festivals, cooking techniques and access to pure ingredients. Diet, digestion and elimination, metabolic energy of *agni* are crucial elements of diagnosis, elements downplayed or absent in “Allopathy” (‘mainstream’ medicine). Its healing practices are informed by customisation at high levels because of its emphasis on *Prakriti* (essential nature, unique to an individual) and personal health responsibility (to be strengthened against *Vikruti*) and importance of location and community life practices (see Lad 2002) Combined with Yoga, it provides a range of life supports that go beyond the physiological. The traditional institutional and organisational mix of modern biomedical interventions is not neatly aligned with Ayurveda’s authority, practice and philosophy.

India’s AYUSH Ministry combines Ayurveda (A), Yoga (Y), Unani (U), Siddha (S) and Homeopathy (H) and also Sowa-Rigpa (Tibetan-Himalayan) each with distinct philosophies and practices and contested traditions with ‘modern’ or mainstream science and clinical practice. These tensions are sometimes with Ayurveda alongside generic and patented pharmaceuticals, with the latter accusing the former of false advertising, overstated clinical efficacy and missing oversight. Soaring consumer demand for Ayurveda during the pandemic exacerbated the friction (The Week 2021). The AYUSH Ministry provides a significant departure from the policies and regulations for synthetic pharmaceuticals and biotechnologies. Ayurveda has a life and medical philosophy, specific techniques that are bundled with services (from ancient surgery to massage), products that have high significance for biodiversity and natural resource protections which are locally bounded (specific oils, plants and extracts, local cuisine, and local food preparation techniques using sustainably sourced and recycled systems) and services tied to new physical infrastructure investments. These include clinics and hospitals, hotels, restaurants, combining health, pilgrimage, medical, and yoga tourism and extending to mental health, sports, medical training and wider education.

‘Authentic’ Ayurveda focuses on ingredient purity, its ecological/regional context (which affects seasonality of physician observation, diet, treatment options), and a customised patient-regimen. These characteristics are at odds with current generics and patented pharmaceutical and biotechnology sectors. More profitable and opportunistic industry segments from pharmaceuticals dominate standardisation requirements:

The quality control methods modeled on those applied to western medicines and phytochemical industry is incompatible and meaningless to ASU industry. Further the present policy has adopted a post-mortem approach, with an intention to reject the products in the event the finished product does not meet the standards laid down in official pharmacopeia. [….] the manufacturers are putting the official botanical entities over the labels but using altogether drugs from different botanical sources, thereby making mockery of the law (Upadhyaya 2011, p.254).

Note: ASU here is Ayurveda, Siddha, Unani medicines (three of those listed in AYUSH).

Despite some industrial manufacturing similarities, Ayurveda has manufacturing elements of different scales and requiring diverse degrees of ‘handmade’, ‘small-batch’, or ‘factory-based’ production (see also Upadhyaya 2011; Harilal 2009; Dejouhanet 2014). Furthermore, different epistemic and ontological contexts define training and practitioner norms. The AYUSH Ministry advised using Ayurveda for immunity-boosting, at-home treatment, or in conjunction with or in lieu of, “modern” medicine, with new attention to clinical observation, assessment, customisation, and its regulatory frameworks^1^(see Vaidya 2011) including randomised placebo-controlled pilot clinical trials for Ayurveda that have yielded positive outcomes (e.g. Devpura et al. 2021; Girija and Sivan 2020; Rastogi, et al. 2020). The importance of case-based medicine and protocols and clinical phasing coincides with a growing body of Ayurveda research during COVID-19. Yet, potential institutional and organisation reconciliation toward health improvements in the two systems has been muddied with competitive slanging, including under the name of religion, medical corruption, and professional incompetence (see Kamath 2021).

This has resulted, despite high demand, in continued high institutional variety as well as policy ambiguity for clinical authority and uninterrupted availability of recognised treatments and medicines. At the same time, more reductive strategies in ‘modern’ science of private and public research labs have led to extracting drugs, cosmetics, and nutritional supplements from Ayurveda (Mittal 2020). Practical problems result: available and routine Ayurveda treatment choices face uncertainty, patchy overlap with hospital care and lack of insurance coverage. Despite high demand for use of Ayurveda in mild and moderate cases (including alongside mainstream medicine, see Mishra 2021, Economic Times 2022) and for boosting immunity as a prophylactic, Ayurveda doctors and suppliers of treatment and formulations encountered a regulatory morass to treat COVID-19 routinely except with individual patient consent and only outside formal clinical settings. With high-profile disputes and controversies about the relevance of standardised, controlled trials, there are unresolved institutional norms and perceived “dilution” of “authentic” Ayurveda in treatment regimens for COVID-19 and queries of the government’s self-care protocols during later waves. A clinical case repository of treatments and outcomes was recently launched by the Ministry of AYUSH, making it more likely that some professional norms of mutual acceptance or mixed treatment strategies will be bridged in the future.^2^ Yet, when high fatality rates from comorbidities of diabetes to high blood pressure needed urgent attention, Ayurveda’s known treatments for these conditions could potentially have played a wider role to contain these in patient-solicited medicine (e.g. Shirkande and Shirkande 2022). International demand also grew during COVID-19: over 18 countries signed agreements for exchange of Ayurveda and other knowledge, products, and services. International Chairs for Ayurveda academic specialisations have also been explored (Economic Times 2022).

While clinical practice and treatment protocols had little resolution, manufacturing concerns predated the pandemic. Ayurveda has both ‘traditional’ and ‘modern’ manufacturing facets, which operate under high institutional variety: e.g. technical standards, certification, quality standards, differing from other medicines. Uncertainty about regulations shapes industrial policy design. Ayurveda’s source products have high purity needs for hyper-localised natural resources such as leaves, flowers, or other parts of plants and herbs. The ability to retain the quality of these inputs is defined by specific types of technological capabilities which have been under-valued. These capabilities must be regionally dexterous, identify and process biodiverse resources, but are currently segmented and fragmented by a patchy certification system, cataloguing problems with authenticity and purity, and dilution of evaluation and procurement standards for manufacturing. Newspaper reports and informal discussions with experts indicate active opposition to Ayurveda by some mainstream medical practitioners and perhaps associated variability in trust by patients.

While one aspect of institutional variety is evident in diverse norms, standards, and regulations between Ayurveda and mainstream medicine clinical practice, the other is the *organisational* variety in the ‘Ayurveda industrial system’, where diverse practices, services, and organisations operate under specific cultural norms, formulations, and internal standards. Some Ayurveda therapies and products are diagnosed, customised and clinically supervised with expertise, others have a mass manufactured standardised clinical regimen and factory product that is closer to pharmaceutical generics; yet others operate with loose regard for safety or efficacy. The Ayurveda ‘industrial system’ has thus remained messily situated amidst ‘traditional’, ‘informal’, ‘cottage’, or ‘home-based’ labels that prevent consolidation and clarity of the structure and boundaries of the industry. It contains a unique knowledge base, valuable ecological resources, patchy supply chains, firms, manufacturing sites and technologies, and accredited medical experts of its own. It needs an alternate lens for ‘sustainability’.

Table 2 includes at least 4 Ayurveda organisations and their networks: ‘Modern’ manufacturing firms, farms and associated nurseries, cooperatives, or other practitioners. Not explicitly addressed here are small pharmacies in Ayurveda which may diagnose and prepare basic remedies and other authentic, highly respected known Ayurveda ‘houses’ which have home-based manufacturing in small-batch, custom-made, and high supervision by doctors or experts in the tradition.

**Table 2:** Institutional variety in Ayurveda manufacturing

Manufacturing institution	“Modern” manufacturing firms	Farms and nurseries	Cooperatives	Other organisations, e.g. small Ayurveda pharmacies	Family-linked production including by TM practitioners
Resource source	Traditional supply chain network	Planting	Planting and harvesting	mSMEs and lead suppliers and distributors often known in the area	Regional specialisation, close to source materials?
Quality control and standardisation	Strong but variable	Highly ecologically variable	Highly ecologically variable	Unbranded and dependent on others	Strong and by family practitioner reputation or associated retailers

A potential expansion of this table would include new reputation-focused procurement systems

*TM* traditional medicine, *mSME* micro and small and medium-sized industries

Quality control and standardisation are both essential in Ayurveda:

Standardization is not a new concept in Ayurveda but is inbuilt in the system itself under the name Dravya Sampath*.* This *Dravya Sampath* is in fact influenced by place of origin, time of collection and administration quality, method of storage and potency enhancements, by virtue of which the drug is capable of producing desired results (Upadhyaya 2011).

However, nomenclature, classification, and oversight are not simply aligned with mass pharmaceutical manufacturing of other consumer commodities. The tensions between the two systems have been reduced to ‘traditional’ versus ‘modern’ industry, but this hides more about institutional variety than it analytically reveals. For example, the concept of Dravya Sampath (standardisation) recognises that microscopic and phytochemical evaluation procedures and priorities for manufacturing and quality standardisation set by routine industrial policies do not fit the needs of Ayurveda, nor its own age-old practices to ensure quality (Ibid., pp. 241–246, and 258–9). While a traditional industrial “raw material” is seen as a simple, unprocessed commodity, Ayurveda inputs involve plants and the biome of the individual in an ecosystem that is known to have complex, systemic features (Ibid.). Other organic matter is included, as are fermentation processes, and industrial sites and facilities. Thus, routine mass manufacturing and standardisation remove several customisation concerns of sourcing and refinement that Ayurveda considers essential. There are now more digital platforms from private sector, some with Ministry supports, for procurement and e-commerce that are standardising availability and search of known, respected brands.

Table 3 further indicates why the “traditional medicine” label itself is unhelpful to industrial analysis and hides the intertwining of organisational variety, disciplinary knowledge and professional skills, each regulated under different institutional contexts.

**Table 3:** Ayurveda and generic pharmaceuticals: standardisation and stakeholders

Types of medicine	Knowledge source	Quality evaluation	Manufacturing process integration	Raw material	Regulations
Generic pharmaceuticals	Chemical (raw materials, chemists and engineers)	Well-established (but gap with customised treatment, results in standardised products)	Well-established	Chemicals and chemical industry	Global and national regulators with clear signalling for most standardised products
Ayurveda formulations	Botanical (raw materials and Ayurvedic doctors, increasingly with chemists, engineers, and biologists)	Lack of standardisation protocols in modern industrial systems to evaluate medicinal plants (strength with authentic products is a highly customised treatment efficacy)	Patchy for smaller brands	Medicinal plants and plant nurseries	Difficulty in large numbers of and fragmented institutions in standardising/Dravya Sampath

Source: author’s elaboration of Upadhyaya (2011) and other Ayurveda sources

Upadhyaya (2011, p.4) further elaborates some systemic gaps:

“1. Lack of control over collection and trade of medicinal plant raw materials. 2. Presence of multiple authorities like Forest Department, Bio Diversity Board, Medicinal Plant Board and Drug Control Agencies of Central and State Governments. 3. Lack of institutionalized regulatory mechanism to regulate medicinal plant trade. 4. The absence of clear cut law to enforce regulation on medicinal plant raw materials trade. 5. Shortage of infrastructure and expertise to achieve the purpose of standardization and quality control of medicinal plant raw materials.”

Unfortunately, the gaps have made easy entry to the ‘Ayurveda’ label, spurring an industry (domestic, multinational, micro to large firms) in medicines, cosmetics, and food products which expand by building and brand advertising of “natural” medicine and consumer products but which may not stand up to authentic expertise. At high demand for Ayurveda for mild and moderate cases and no doubt some to avoid draconian hospital isolation protocols, patients appear to have used specialised suppliers as well as mass market food and cosmetic companies such as Dabur and Himalaya, not just for COVID-19 mitigation treatments (products and services) but also immunity-boosting formulations and natural herbs, fruits, roots and spices, respected with *dosha*, seasonal, and disease-specific oils and formulations. Mass market companies such as Brihat, Baidyanath, Dhootapapeshwar, and Kotakkal Arya Vaidyashala, alongside smaller Ayurveda 'houses' need categorisation relative to those self-termed ‘Ayurvedic Pharma’ companies, e.g. Amrut, Revinto, and Sri Chamundeswari.

However, Ayurveda is experiencing greater demand as a lifestyle health preference with growing scepticism of mainstream medicine, such as worries about side-effects, focus on symptoms but not cures, severe isolation of patients from families during COVID-19, and rising costs. Ayurveda’s demand had risen further because of ageing global populations, and India’s own rise of non-communicable diseases (NCDs) and lifestyle linked diseases, low perceived toxicity and side effects, combined use of Ayurveda with yoga, emphasis on boosting energy, immunity, respiratory and mental health. Yet, individual Ayurvedic practitioners struggle to maintain a business and the system’s quacks may be tempted by the same regulatory gaps, generating potentially harmful substances and undermining the reputation of a systematic body of alternate scientific knowledge. The foundations of the Ayurveda industrial enterprise have arguably been stripped of their epistemic base and muddying of customary use. Despite demand, rural consumption is shifting, facing inexperienced practitioners and uneven product quality. Clinical practitioners themselves face competition and may be tempted to dilute their systems of knowledge by offering “one-shot” solutions (injections, tablets). There is anecdotal evidence from specialists that, in order to mimic or compete with mainstream medicine, Ayurveda practitioners may offer higher dosages or easily available but poorer quality formulations to secure desired health outcomes. There are additional pipeline problems, such as skill challenges and uneven quality of medical personnel (although some specialists contacted for this study indicate that after a period of decay, things are now improving with capable students finding Ayurveda a viable professional option and healing vocation).^3^

The tensions within Ayurveda and externally with pharmaceuticals and biotechnologies show a battle for appropriate certification and contemporary standardisation norms and policies and a hollowing out due to neglect of mass manufactured and branded products which use a cover of ‘natural’ authenticity but arguably cannibalise Ayurveda’s core. Cultivation and protections of forests and practices associated with medicinal plant nurseries are also associated with lifestyle such as food, different tribal and religious or spiritual traditions. It also serves a profitable ‘eco-friendly’ tourism, and especially after COVID-19, some departments, clinicians, and researchers of mainstream and Ayurveda systems work alongside, addressing immune response, osteoarthritis and Parkinson’s disease (e.g. Times of India 2018). Transdisciplinary, Ayurveda and Yoga universities also collaborate. 

Growing demand does appear to increase student enrolment and policy interest but challenges persist. The Indian government has established the WHO centre, boosted Ayurveda exports, and simplified the Harmonised System of Nomenclatures (HSN) codes for harmonising tax and production, goods and services. Yet, the global health architecture remains heavily tilted toward mainstream norms and regulations, quite primitive in acknowledging the sector’s institutional variety and unique needs for ‘sustainable’ industrial policy. The WHO’s own regulatory guidelines for traditional medicine (WHO 2002) do not have routine biodiversity debates or exchange with other standard World Trade Organization (WTO) conventions. The new WHO Global Centre for Traditional Medicine in India offers no further clarity: “Maximizing potential of traditional medicines through modern science and technology”. The WHO GCTM instead uses nomenclature of TM below that confuses both traditional and modern in the same paragraph.

The term traditional medicine describes the total sum of the knowledge, skills and practices indigenous and different cultures have used over time to maintain health and prevent, diagnose and treat physical and mental illness. Its reach encompasses ancient practices such as acupuncture, ayurvedic medicine and herbal mixtures as well as modern medicines.^4^

Some policy changes thus require mutual WTO and WHO engagement to ensure that ‘traditional medicines’ (Indian, Chinese, other) are brought into the wider scope of economic policies and narrower industrial policy, garner geographic indications, and promote future biodiversity conventions. This policy redesign must acknowledge fundamental scientific differences within industrial sub-systems some of which cannot be simply bridged, alongside standard-setting coordination from accredited Ayurveda specialists and micro, small and medium enterprise manufacturer networks.

Case 2 below of oxygen in the COVID-19 environment underscores a different set of concerns of institutional variety, in the contested and competitive environment in which clinical services were delivered and regulatory roadblocks and successes experienced in attending to oxygen shortages.

### Case 2: the ‘industrial vs. clinical’ oxygen case

Industrialised countries also witnessed the pandemic’s oxygen crisis: scarcities of stored oxygen in hospital, what was available to patients through piped building oxygen, canisters of oxygen available to inpatient and outpatient, and other forms of oxygen scarcity. Three large US states—New York, California and Texas—experienced this crisis (Toner 2021). The US initiated a possibly slower industrial ‘wartime’ response, to what was initially perceived as a more manageable regulatory split between hospital sites and management codes for piping of oxygen, and volumes of oxygen storage on site. US oxygen emergencies on site led to a steep rise in critical waiting times, long queues, and special instructions for priority patients (Sutton 2021; Leatherby et al. 2021; Weber 2021). By January 1^st^ 2021, 4374 people with COVID-19 in the US had died in the prior two weeks, 585 deaths on January 1^st^ itself, and 26,000 deaths in California alone. A “brutal directive from LA health authorities to overwhelmed ambulance drivers has revealed the terrifying truth about America’s COVID battle”, with ambulance crews directed neither to give transportation priority to cardiac arrest patients whose survival was deemed unlikely and not to give supplemental oxygen to those whose saturation levels fell below 90% (Weber 2021). The industrial failures of US oxygen support were exacerbated by a major electric grid collapse in cities in Texas. COVID-19 survivors in Texas were sent home before full lung recovery and depended heavily on a variety of electricity-dependent equipment including plug-in breathing machines, oxygen concentrators, bilevel positive airway pressure (BPAP machines) used for several medical conditions, and portable oxygen tanks (Hixenbaugh et al. 2021).

In India in contrast, major cities in the second wave—Delhi and Mumbai—were in the forefront of a continuous media glare documenting patient suffering and inside-hospital coverage, arguably violating patient privacy norms (Chellaney 2021). As with Ayurveda, significant politicisation of oxygen availability resulted in claim and counter-claim, from politicians, administrators, businesses, and NGOs (News18 2021, Times of India 2021a). The spotlight was understandably on clinical failures rather than rapid institutional adjustments in a very short period.

The scope of a single institutional framework for the ‘oxygen industry’ is impossible: oxygen is a feeder industry into a range of influential growth sectors from food to petrochemicals. Box 1 indicates the range of industries and specialisations involving oxygen. Oxygen’s centrality to multiple industries meant that national government coordination was needed to prevent a severe economic slowdown beyond the pandemic’s own effects.

**Table:** 

Box 1 Oxygen’s industrial footprint.	
• “Clinical” oxygen, piping, storage and warehousing, freight and transport logistics, delivery business models-at user (patients, hospitals)	
• “Industrial” oxygen	
• Petrochemicals	
• Oil and gas	
• Steel	
• Highways, flyovers, airports, bridges	
• Retail, construction technologies—new materials, low carbon	
• Cryogenics, helium, neon, and other gases	
• Transport, recharging, fuelling stations, railways, trucking	
• Aerospace, defence	
• PSA on-site energy conversion	
• Battery storage and solar charging	
• Food storage, food processing	
• Chemicals safety, fire safety, technical standards	
• Agriculture perishability and food labs, warehousing, freezing	
• Technical standards, regulatory personnel	
• Security logistics, law and order	
• Repair, repurposing, site-servicing, maintenance contracts, ESCOs	

Source: author’s elaboration.

Industrial oxygen sources were thus initially slow to re-steer to medical oxygen as regulatory barriers (industrial vs. medical oxygen) required new institutional combinations (procurement policies, competition rules, safety standards, transport strategies). Yet within a short period by industrial standards, the oxygen production and delivery was being coordinated between central and state governments (some with populations greater than Brazil, Mexico, France or Germany), channeled by private procurement into larger hospitals, and including off-site innovations. Vocal scientific and political opposition to the working of ministries notwithstanding, and despite hoarding and corruption in private sector and municipal governments, many locations still witnessed improved just-in time delivery of oxygen to patients.

Large private sector companies ramped up production.

India’s largest liquid oxygen manufacturer of the time, INOX Air Products, was a major contributor to the response. Director Siddarth Jain stated:

Do you think India could not have anticipated this spike in Covid-19 cases and no preparation would have been enough?

Absolutely… Why cases spike in this manner is beyond me. I don’t think any country would have been prepared for this kind of acceleration (in Covid-19 cases), which is currently the world’s highest.

All I can say is from an infrastructure perspective, we are scaling up. India has increased its oxygen manufacturing capacity by 30% within a month. This is unfathomable. I don’t think it has happened globally. It’s a world record in itself. The effort has been so enormous, however, increasing the capacity of manufacturing is not the same as getting it to the patient (Sharma 2021).

Despite several patient-end learning and mistakes, a mammoth war-like operation of oxygen ramp-up and delivery was soon underway, involving production successes and enormous geographic logistical planning to move oxygen from industrial sites to non-producer states. From early to late May 2021, the Indian Railways delivered more than 20,000 million tonnes of emergency medical aid (Table 4).

**Table 4:** Industrial oxygen production features common to India and the US during COVID-19

Industrial product feature	Notable Indian use	Notable US use	Where both countries struggled
Oxygen-related equipment and last-mile instruments such as tanks, regulators and liquid oxygen delivery trucks	Extensive use of Indian Railways to transport dedicated LMO storage tankers	Existing road use with plant-level storage	Cylinders and piping
Policies, regulation, personnel	Rapid use of centralised government ministries, acts, regulations and decentralised stakeholder participation to ramp-up production; use of public sector, defence forces, national rail and road, Army Core of Engineers, Defence Production Act or other central government procurement	More decentralised governance, except for invocation of Defence Production Act	
Storage and delivery at hospital sites and beds	Limited oxygenated ICU beds New guidelines for hospital self-reliance	Assumed oxygenated bed but serious mishaps with grid collapse	Tubing for multiple patient beds Home patients and oxygen supply
Clinician guidelines of per-capita use and conservation	Audits and nozzle-flow monitoring provide some gains		Countrywide operational guidance for technical staff to monitor and lower oxygen over hours to reduce per-capita usage (good practice exists in both as well)
Home-based guidelines and access	Several innovations to be studied: providing oxygen cylinders to those ill or recovering at home, including oxygen concentrators, with new business models, purchase, rent, ‘Uber-like’ sharing, etc	Oxygen saturation measurement and simple diagnostics to ensure at-risk patients are admitted where possible	Countrywide home monitoring, quotas or rationing

Source: author’s research

*LMO* liquid medical oxygen, *ICU* intensive care unit

300 ‘Oxygen Expresses’ (trains) took essential oxygen to 39 cities across 15 Indian states (traversing a population spread greater than most South American countries and European regions). The expresses collected industrial oxygen and, through arduous journeys and multiple transport and storage partners, delivered onward to towns and villages, combining new oxygen storage and road transport logistics for liquid oxygen. States with existing industrial oxygen supplies and manageable COVID-19 were able to share with states and metropolitan and smaller cities (industrialised or not) with little or no reliable oxygen supplies or high COVID-19 case counts (Thadhani 2021, Swarajyamag.com case coverage and Government press releases) (Table 5).

**Table 5:** Industrial diversification in the 2 cases

Industry case	Industry type	Related input and output industries and opportunities for further diversification
Ayurveda	‘Traditional’, ‘alternative’	Food, packaging, agriculture, biodiversity, logistics, chemicals and pharmaceuticals, tourism, cosmetics, ‘digital’ and other ‘wellness’
Oxygen production and delivery	Industrial	Steel, construction, railways, trucking, small freight (autos, taxis), robotics, storage and warehousing, device design, tubing, repair and servicing

Source: author’s elaboration

The institutional re-steering of industrial policy was evident in fast-moving centralised and state-level coordination decisions of pricing, procurement, intellectual property, subsidies and new investment, all occurring in a highly compressed timeframe. Most resulted (with controversy and contestation) in alternate institutional rules, from pressures from patients’ families to create new regulations, clinicians and firms seeking clarity on technical standards and equipment design criteria, or in the case of hospital fires breaking out on site, with new building codes and industrial restocking and storage requirements rather than older hospital administration from pre-pandemic times.

Production ramp-up and policy shifts of procurement were underway, with meetings with oxygen producers to ascertain capacity, central and defence help, and new funds to be allocated for Pressure Swing Adsorption (PSA) generation plants. These led to 32 states and union territories and aimed at all districts (Office of the Prime Minister of India 2021).

A total of 1,224 PSA Oxygen Plants have been funded under the PM CARES across the country since the advent of the Covid-19 pandemic. Out of that more than 1,100 plants have been commissioned, providing an output of over 1,750 MT oxygen per day.[..] The project to commission a PSA oxygen plant in each district of the country was executed while dealing with complex challenges of hilly areas, islands and territories with difficult terrain. Around 7,000 personnel have been trained to monitor the operation and maintenance of these plants. These plants will be monitored with an embedded Internet of Things (IoT) device to check real-time functioning and performance through a consolidated web portal (New Statesman 2021).

Both the US and India managed new institutional combinations and activated national defence production acts as needed for vaccines, Personal Protective Equipment (PPEs), oxygen, India with perhaps the faster and speedier cumulative rollout. Diverting oxygen from industrial to clinical use affected several industrial sectors from petrochemicals, to automotive and space research (Shandilya 2021; Jestin 2021; The Hindu Business Line 2021). Both public sector organisations (oil, defence, power) and private conglomerates such as Reliance, Adani and Tatas diverted supplies by scaling down or ceasing other industrial operations, lowered prices, including some free provision (see Thadhani 2021). Private firms took losses but also invested in future demand. Private firms such as INOX moved quickly in oxygen but also approximately $268 million in Indian greenfield investment for industrial gases, Air Separation Units (ASUs) and operations in 44 Indian locations, expecting high demand for nitrogen, argon and helium (used in healthcare, automotive, chemicals, semiconductors) and investments in seven major industrial corridor states (Mukul 2021; OmmcomNews 2021).

Both private sector and public facilities and companies were consulted. National defence, private industry, and public, private, and hybrid organisational arrangements with new centre-state coordination manifested in new rules since health is traditionally a state-level subject in non-pandemic times. Institutional variety—from norms to technical standards, unexpected consultations and commitments to national interest, invoking existing laws, introduction of new laws, all reoriented availability of oxygen to patients and across industries (see Srinivas 2021a, b, c, also Graham and Falade 2021). PSA systems were set up speedily but whether they are reliable at scale remains to be judged. Yet, the Indian oxygen expansion demonstrates that new institutional combinations—from laws to procurement, logistics and freight, new technical and fiscal norms, can in fact be addressed under dire conditions.

Low IV exists in the standardised industrial component of oxygen production, but high IV is visible for customised patient bedside, home, or refilling delivery services. For example, at-bedside in Mumbai’s dense hospital contexts provided useful information on improved technical specifications of flow rates and fixes (Times of India 2021b, c). There remained many serious challenges and tragedies on-site at hospitals from restocking, refilling, and fires. Flow pressure requirements for patients, fire safety storage, and regulation of hospital sites and building codes will likely emerge only from more systematic evaluation of operational experience (for failures, see Hindustan Times 2021, Scroll 2021). Confusion of demand estimates and storage challenges on-site at hospitals and clinics reveals the mismatch of industrial policies with health policies in practice (such as faulty building codes and cumbersome restocking rules). The skills required for industrial site and logistics management which apply at large oxygen facilities are far less rigorously followed at smaller hospital sites. Public administration was crucial (e.g. The Better India Community 2021). The PSA fiscal disbursement and commission however forced lower-level authorities to manage the resources, fixed investment, changing facilities regulation as well as management and repair systems. At-home services lent themselves to dense community networks and new business models which brought technological innovation (Muthukumar 2021; Nitnaware 2021).

## Preliminary observations

This paper contributed two brief industry cases sourced from fast-changing news reporting, policy statements, early clinical and scholarly publications, and business briefs during the pandemic. A much deeper analysis and systematic research design is needed to take on this complex topic. The goal here was to observe how the pandemic’s compressed timeline affected institutional change. At a minimum, the analysis permits debate about the goals and process of “sustainable” industrial policies (SIPs).

First, there are evidently different degrees of institutional variety (IV) that can co-exist in a single country under high demand and uncertainty, each with varying degrees of success and judged by different criteria. Arguably, higher IV with greater policy ambiguity has resulted in challenges for Ayurveda because it poorly fits the generic pharmaceutical model of high standardisation with low IV and has a unique scientific and knowledge base including dependence on high-quality natural resources. High patient demand makes its SIP case urgent. Low IV exists in the standardised industrial component of oxygen production but high IV in at-bedside oxygen led to several innovations for patients. Oxygen’s crucial role in multiple industries makes its SIP case urgent as well.

Future studies should ideally further focus on partial ‘success’ or experimentation of new institutional combinations: e.g. legal challenges of Ayurveda medicines, innovations in small *n* patient case management, last mile delivery innovations in oxygen, high speeds of industrial oxygen production ramp-up, and defence-civilian-private sector collaboration. Defining the boundaries of Ayurveda’s or oxygen’s capabilities and related industries determines which norms, customs, technical standards or regulations govern the design of industrial policies and will fine tune relevant SIPs. Both cases have ‘small batch’ production and at the same time relate to large, profitable industries. Both have unregulated sectors which are poorly standardised for health and safety (e.g. cosmetics in the case of Ayurveda and industrial sites, building codes and storage regulations for piped oxygen). In oxygen’s case, the standards and rules that govern transport, storage, building codes, stocking and tubing are in conflict across many countries between ‘industrial’ oxygen (meaning largescale industry) and ‘medical’ oxygen, seen as regulated through hospital facilities and associated sites and services, which have not traditionally had to consider the volume of oxygen stored or the diverse means by which it has to be delivered to patients on site or in home isolation.

In this sense of institutional variety, more formal ‘rigid’ industrial or medical standards of industrialised economies would have been unlikely to quickly adapt in a pandemic. Higher IV may lend itself to greater innovation but also greater uncertainty for organisations. It will take further research to determine if Indian adaptability and new business models are better at combining and coordinating both micro-scale delivery with larger scale centralised coordinating and planning. The US’s preliminary evidence of oxygen crises—from weather to collapse of feeder grids for electricity, and its own safety standards appear to indicate some continuing vulnerabilities in serving patients even in highly industrialised economies. Ayurveda on the other hand appears to have a core of systematic theory and practice, but whose institutions of standardisation and quality control of essential plant resource inputs have been under continuous stress for decades. Furthermore, the WHO’s TM strategy (2002–2005) (WHO 2003) is focused on institutional design of finished products and therapies but not their industrial process: issues such as certification, regulation are focused on, but not the sourcing, process manufacturing, or early certification and regulation of source products (Srinivas 2021b).

Long-term sustainability of oxygen’s industrial potential lies in cross-industry regulation, selective centralisation, and close industry partnerships at both large-scale production and demand management, but still require site-based and neighbourhood-based last-mile regulation of delivery systems.

In both sectors, there are practical technological capabilities missing: talent and staff training, logistics from source sites to patient, storage to transport reliability, and considerable gaps in quality and safety certification. Both require new point-of-care approaches and patient choice, combined with technical facility of instruments and storage units, including design and use, repair, refilling, and restocking. In the Ayurveda case, source materials being crucial and scarce, more stringent national biodiversity protection aligns well with the industrial aim for quality, safety, and efficacy.

## Discussion: institutional variety and sustainable industrial policies

The article began by asking under what conditions industries were sustainable and provided two brief cases with high demand and high uncertainty in the COVID-19 pandemic. These provide important qualitative although preliminary case material on institutional adaptation under highly compressed timelines. At a minimum, the two contrasting cases indicate that institutions from norms to rules are indeed mutable under stress and operate under diverse and perhaps ill-fitting policy. At its maximum, the IV approach can make more precise a set of hypotheses about new taxonomies for SIPs and their forms of combination, selection, and evolution over time. And no matter whether low or high degrees of institutional variety exist, societies may be unable to combine them in ways that are technologically viable with their existing capabilities or business models.

Five implications of IV and SIPs are briefly discussed.

### Can there be a “sustainable” health industry without ecology?

The current and next decades are crucial for ecological sustainability. The question raised, “when is industry sustainable”, can be answered first by acknowledging the dynamic of combinatorial logic of effective industrial and regulatory policies to manage more than one health sub-system and their ecological co-existence. For instance, the UNIDO (2022, xvii) definition of inclusive and sustainable industrial development (ISID) is “Long-term industrialization that drives development along three aspects: creating shared prosperity by offering equal opportunities and equitable distribution of benefits to all; advancing economic competitiveness; and safeguarding the environment by decoupling the prosperity generated by industrial activities from excessive natural resource use and negative environmental impacts.” In the realm of actual institutional variety, the two cases could be described as higher IV (Ayurveda) and lower IV in industrial oxygen, but with higher IV in customer segments. Neither satisfies the normative UNIDO goal, but the taxonomy of IV we have seen across the tables points to the need for clarifying the goals of industrial policy and aligning industries in the direction of healthier populations overall.

Thus, a major gap and opportunity exists within EPE to assess both meso- and micro-heterogeneity that better combine ecological, health, and industry concerns. This requires a fundamental shift from normative and vague policy ideas toward real adaptation evidence. Ayurveda and industrial oxygen production are both elemental in their claims on natural resources, whether botanical, synthetic, biomedical, or industrial scale. The growth of accredited Ayurveda in its purest forms is arguably well aligned to fundamental tenets of sustainable growth and SDGs, enhancing biodiversity and natural health resilience of individuals and communities. ‘Sustainable’ can also be viewed as systemic institutional design to correct those efforts which are resource-intensive or industrially ill-advised. In some cases, intellectual property rights combined with compulsory licensing or new business model innovations may simplify at-home care, improving norms of low-cost, fair and humane use and respecting patient choice. This approach to IV helps dynamically frame *health risks alongside their industrial foundations* (see also Srinivas 2021b). Combining health, ecology, and industry together makes the term ‘sustainable’ more amenable to analysis and to diverse system philosophies such as individual responsibility in health and biodiversity protections.

### Addressing long-term costs and savings in health

This explicit attention to institutional variety is necessary to reflect on categories of economic activity and their health and environmental repercussions. Ayurveda’s focus on health and not disease emphasises sustenance, preservation, and living closer to nature and stresses personal responsibility, energy use and health. The logic of Ayurveda and its emphasis on respiration, immunity, energy-enhancing practices treat human health as a part of their ecological present. It requires new types of industrial policies and commitments to health education and health services that minimise non-communicable diseases and mental health or other disorders. As the pandemic has revealed, loss of biodiversity, habitat, and local ownership of resources can combine to undermine health and food security, heightened zoonoses, and other climate challenges such as flooding and extreme heating. Here, the ecological view of Ayurveda has promise.

For most low and middle countries, the dominant form of payment for healthcare is also out-of-pocket payments, not insurance, and through sizable private sector service delivery (both business and non-profit), also shaping economic burdens of costly side effects and treatments of ‘mainstream medicines’ and faulty, poorly regulated ‘traditional medicines’ alike.

The ecological siting of individuals, their personal responsibilities and choices, and the systemic analysis of health thus vary across the two cases. The temporal dimension of health is further different, with the emergency of the pandemic creating uncertainties about which types of interventions to pursue and with oxygen required in an emergency and Ayurveda perceived as essential to long-term immunity.

Exploration of institutional variety to its full combinatorial extent can therefore be useful to consider which types of healthcare—not merely those clinical or biomedical domains—might be combined and provide economic development and cost-saving gains in the long term. These may refine our contemporary notions of public health, community health, inter-generational responsibility, and individual responsibility alongside. For evolutionary political economy, these axes of consideration of new types of costs and benefits may lend themselves to more precise methods and policies.

### Economic classification

Ayurveda has different scientific and individual and specialty practices, some of which convert to services. Its products and services and its own ideas of authority and tradition create a system of ecology, manufacturing, science and medicine that may need different regulations. In it, health choice and healthcare come bundled with life practices, individual decisions, self-awareness, products and services (whether from own-garden products, or sophisticated knowledge practices), specialists and training (minimum Bachelor of Ayurvedic Medicine & Surgery (B.A.M.S.)). Also included are ‘off-the-shelf’ medicinal ointments, tablets, liquids, or healing techniques.

Classifying economic activity in the ‘heath industry’ has many challenges, only briefly discussed here. (i) First, and perhaps most importantly, it is difficult to frame an individual’s own physical, monetary, or time investment in their health and difficult to study with respect to outcomes, within and certainly across medical systems. (ii) Second, health systems differ widely in their epistemological and ontological foundations that place human, animal, and plant health as essentially intertwined. This may make industrial ideas of scale and scope impossible to apply as originally imagined in steel or automotive. (iii) Third, the industrial foundations of any healthcare system—such as manufacturing, technology, and knowledge protections, are usually underestimated, whether ‘traditional’ or ‘modern’, and with digitisation is changing all of these.

Therefore, ‘services’ of ‘traditional medicine’ thus do not follow what is usually a biomedical economic model of the ‘health industry’ for at least 3 different reasons: Such services (a) bundle self-care and personal responsibility as relevant economic actions and economic investments (which potentially have temporal and preventative savings of magnitude including but not limited to mental health), (b) include practitioner services as ‘clinical’ authority with prescribed ‘health services’ (the more routine understanding of practices which may extend from massage to home health services, or geriatric care) and (c) provide services understood as built within or adjacent to specific industries and which may be technologically and digitally growing (includes a rise in private sector activity in telehealth, diagnostics chains, and sectors such as specialised expert consulting from ergonomics to materials testing). Note that Ayurveda originates in (i) and (ii) above but may be amenable to (a), (b), and (c), in marked contrast to traditional ‘modern’, ‘Western’, ‘allopathy’ which may excel in emergency surgery for example. Finally, a unique category of services (d) that are compatible with (i) and (ii) as well as (a), (b), (c) and is environmentally and biodiversity enhancing. These may include new farming, local source harvesting and packaging, education, indigenous certified Yoga, tourism and hospitality services, purity of materials and formulation consulting, and digital platforms for consultation and delivery of bundled AYUSH care. This category of services and manufacturing-related services can steer toward situating human health firmly within climate and biodiversity responsibility. Oxygen production and delivery services have their own potential ecological impact which would be useful to study. The routine division of industry and services can use Ayurveda and oxygen to explore more useful economic classifications of health-enhancing and ecologically-oriented services.

### Standardisation policies and new business models

The rise in larger firms and research labs engaging in ‘parasitic’ or ‘symbiotic’ R&D and foraying increasingly into biological resource materials and Ayurveda knowledge implies that, for co-existence, a type of industrial protection for Ayurveda could be considered to retain and strengthen its knowledge foundation: source materials in Sanskrit, regional languages, documenting of scientific methods, training of practitioners from medical experts to manufacturing specialists, and finally ensuring input quality, especially to manage sophisticated source and supply chain methods for specialised medicinal plant nurseries (including fast-disappearing plants).

Thefore in Ayurveda, despite high IV, focused industrial policies can.

- boost traditional regional specialists and nurseries
- launch wider geographic indication (GI), geo-tagging and biodiversity of plant nursery supply chains
- multiply sources and procure improvements in small-batch and just-in-time manufacturing systems through third-party accreditation and certification systems of “authentic” Ayurveda
- promote regulatory quality process improvements and focused patenting

These localised but wide-platform enhancements can benefit most firms, farms, and nurseries. Similarly, oxygen demand and delivery models can prioritise businesses that need to deliver small cylinders, rapid refill, and delivery on bicycles or motorcycles or which build on digitalisation of emergency health and location data. Those which collect and refill at the touch of a mobile phone are more likely to be useful and adaptable to small firms with low market -entry thresholds. Similarly, where traditions of self-help, home care, lifestyle change, or highly customised diagnosis through Ayurveda is the norm, small batch manufacture and traditional, respected, authentic ‘cottage’ manufacture will be respected much more than a commercial ‘brand’. Purely scale of manufacture and shelf-life considerations may get lower priority.

As COVID-19 has shown, a point of friction is evaluative criteria for customisation and patient outcomes. Ayurveda manufacturers will have to adapt to new strategies for evaluation of quality, safety, and efficacy but also protections of rare and pure source materials. Procurement rules should be a first point of industrial policy efforts. Moreover, competitors cannot regulate or set standards for others. Mainstream medicine and Ayurveda cannot be regulated exclusively under rules set by the former. Finally, there may be merit to protecting some domestic industry sub-sectors from too-rapid integration and standardisation. Global integration requires harmonisation for lowered costs and higher revenues, but prevents domestic customisation. In order to be ‘sustainable’, industrial policy has to permit firms to service their local markets and adapt to diverse local production and regulatory concerns. This is especially true for large domestic markets such as India and China but also for exports into heavily trade-harmonised regions such as Europe or the US.

### National and industry sub-systems matter to economics methodology

Institutional variety maps how we define systems and their evolution. There is no single national system for health in India, and Chinese traditional medicine may provide a non-democracy contrast for IV. Comparative studies are needed to understand if industrial sub-systems are ultimately reconcilable. Detailed case studies of ‘small’ or micro- and meso-scale institutional variety, whose technological capabilities explain why and how IV is narrowed or widened, can lead to sensible evaluation timelines for sustainability.

Because billions worldwide depend on ‘traditional medicine’ combined with other sources of mainstream routine and emergency care and services, it remains critical to improve affordability, quality, reliability, and safety of such *combined*, *co-existing* systems as well as their industrial mismatches—from regulatory oversight, sourcing differences, certification systems, and alternate systems of authority. Of course, more systemic policy interventions (beyond industry) are needed, e.g. early health and geography education, low cost but ecologically sustainable local food and other source materials such as oils, minerals, and rare plants, and combined health approaches (e.g. mind–body such as Yoga, Ayurveda-Siddha).

Institutional variety in EPE becomes a useful first step to recognising economic systems and their transitions. The IV combinations and the technological capabilities they require can reveal new models for combined public oversight through detailed qualitative case studies within nation, within industry, and across global health efforts. It is useful to recall that economics’ reliance on mathematics and particular use of linear algebra generated the marginalist revolution toward resource use and welfare theory that later shaped health economics. Institutional variety suggests that a different combinatorial algebra from qualitative analysis of suitable cases may help move the discipline forward again. A sole emphasis on ‘clean’ longitudinal datasets in healthcare to run econometrics or to assess regulations will have limits in inference and policy judgement.

## Conclusion

Industrialised economies and their rules dominate global trade, but are not necessarily well suited for ecology or climate considerations, effects that they might even have precipitated. This article argued that institutional variety (IV) provides a proxy for institutional and organisational combinations that can be especially helpful to track in countries with existing technological capabilities and which are sufficiently industrially diversified. These can benefit from revisiting intra and inter-industry linkages and organisational variety. Tensions between their existing industrial policy instruments however prove costly if unattended: new ways of combining intellectual property, competition policy, cooperative and community behaviours to manage resources, or more attention to the co-existing industrial sub-systems which can thrive alongside and hidden capabilities brought to bear.

If lower institutional variety (IV) is a specific policy goal for any country to build cohesion amongst proliferating and conflicting institutions and organisations, this should be explicitly acknowledged in industrial policy design. Too-early standardisation or regulation that lowers IV sharply may do more harm. The relationship between high IV, innovation and adaptation needs analysis. Nations and industries that have been seen as sub-optimal (“developing”) may well have retained some combinatorial agility, and those “developed” with standardised care may have been unable to suitably respond to patients.

As laid out in the introduction, clarity on SIPs is essential to any industrial policy reform, especially as nations struggle to harness opportunities in the post-pandemic and war context of high inflation and interrupted supply chains. Of special note will be energy transition policies’ effect on health system design especially in reshaping agriculture and natural ecosystems. Strong state coordination of private actors, differently regulating emergency and long-term health services, and wider approaches to cross-industry, services, and agriculture SIPs is needed. This balance and recombination of strategies between low and high IV is a preliminary theoretical lens for evolutionary political economy debates on variety to build a real-world taxonomy of viable SIPs over time.

## Footnotes

1 See: https://www.ayush.gov.in/, from the Ministry of Ayush for several suggestions for preventative remedies and amelioration strategies (last accessed 4 June 2021). The Confederation of Indian Industry also has an Ayurveda group. See also CII (2021).

2 See Kaul (2021): “’The Ayush Clinical Repository (ACCR) portal (https://accr.ayush.gov.in/) will serve as a platform to support both Ayush practitioners and general public. This portal aims to aggregate information about clinical outcomes achieved by Ayush practitioners on a large scale. It will facilitate not just dissemination of information but also further analysis and research. It is expected to document the strengths of Ayush systems for treatment of various disease conditions,’ said Ayush ministry in a statement.”.

3 Drawn from personal communications with several Ayurveda clinicians in 2020–2021.

4 https://www.who.int/news/item/25-03-2022-who-establishes-the-global-centre-for-traditional-medicine-in-india, last accessed 12–08-2022.
